# Neck-MRI experience for investigation of survived strangulation victims

**DOI:** 10.1080/20961790.2019.1592314

**Published:** 2019-05-07

**Authors:** Christine Bruguier, Pia Genet, Jean-Baptiste Zerlauth, Fabrice Dédouit, Jochen Grimm, Reto Meuli, Tony Fracasso, Silke Grabherr

**Affiliations:** a University Center of Legal Medicine Lausanne – Geneva, University Hospital Lausanne, Lausanne, Switzerland;; b Department of Diagnostic and Interventional Radiology, University Hospital Lausanne, Lausanne, Switzerland;; c Clinique Cécile, Lausanne, Switzerland

**Keywords:** Forensic sciences, forensic pathology, neck-MRI, radiological findings, medicolegal evaluation, strangulation survivors

## Abstract

For the medicolegal evaluation of victims of survived strangulation, a neck-magnetic resonance imaging (MRI) can be performed for assessing lesions in the inner soft tissues (fat, muscles or lymph nodes, for example). In our institute, such MRI examinations have been performed for a test period of 4 years with the aim of evaluating the use of this tool by forensic pathologists and identifying medicolegal indicators for the performance of neck-MRI in surviving victims of strangulation. We retrospectively reviewed medicolegal reports from all victims examined during the test period. We extracted objective lesions (e.g. petechiae, bruising and abrasions) and reported clinical symptoms (e.g. vision disorder, dysphasia) from the reports. These findings were compared to those reported from the neck-MRI. In total, 112 victims were clinically examined after suspected strangulation. Eleven of these victims underwent an MRI examination of the neck. Eighty-four of the victims presented objective lesions during the clinical examination, with eight showing signs of both petechiae and bruising. Neck-MRI was performed in four of these eight victims and three of them showed lesions visible in MRI. Of 76 victims with bruising as the only objective finding, 66 victims described clinical symptoms. Of those 66 victims, seven were examined by MRI and two demonstrated lesions in MRI. When MRI was performed, relevant findings were detected in 45% of the cases. This leads to the suspicion that many more findings could have been detected in the other victims, if an MRI had been performed in those cases. Our results lead us to the conclusion that an MRI examination of victims of suspected strangulation is useful, and strict indications for its application should be established.

## Introduction

In forensic medicine, the examination of victims of survived strangulation is an important medicolegal issue [1–4]. Forensic pathologists have to assess the assault and the most important challenge is to evaluate whether the strangulation was life-threatening. Thus, an external clinical examination has to be carried out with the aim of identifying and describing lesions such as the presence of petechial bleeding in the face and neck, bruising to the neck and/or skin abrasions. Besides such objective signs, a detailed anamnesis of the victim seeks to search for clinical symptoms that are then taken into account for the medicolegal evaluation of the case. Such subjective signs include the description of symptoms related to the victim (e.g. signs of cerebral hypoxia, such as loss of consciousness, loss of urine or feces), as well as symptoms reflecting possible inner lesions such as dysphasia, changes of the voice and pain while swallowing. At the end of this clinical examination, the forensic pathologist should confirm or reject the life-threatening status of the strangulation, taking into account objective evidence and the clinical symptoms described by the victim. Currently, forensic pathologists can only rely on the scientific literature [5, 6] and their national forensic society and its recommendations to come to their final conclusion. Furthermore, such recommendations differ from one country to another; even for the most important forms of evidence (e.g. presence of petechial bleedings), there is no international consensus to declare whether an event was life-threatening or not [7, 8].

In recent years, the performance of a neck-magnetic resonance imaging (MRI) examination has been proposed to document lesions which are not visible during clinical examination more objectively [9, 10], and to support forensic pathologists in their interpretation, especially in cases where the event is considered to have been “life-threatening”. Indeed, MRI is a powerful tool for investigating soft tissue and therefore appears well-suited for analyzing the soft tissue of the neck [11, 12]. Initial studies have shown the advantages of such an additional exam for medicolegal documentation and the interpretation of findings [13, 14].

Convinced by the results of these preliminary studies, one of the forensic pathologists in our institute set up a research study proposing a neck-MRI for the examined victims of survived strangulation. To support and compare the results of the aforesaid studies, the inclusion of victims presenting petechiae, bruises and/or abrasions in the neck and/or in the face was proposed in order to enhance objective evidence. It was also proposed to include victims without objective lesions based on the presence of clinical symptoms. The forensic pathologists of our centre had an information session about this study. The existing scientific literature and the procedure to access the MRI device were provided to them. There was no order from the head of the department for the use of this medical examination, thus based on these indications mentioned above, their knowledge of the potential added value of the MRI and their own experience, the forensic pathologists had the option of adding this supplementary procedure to their medicolegal evaluation.

During a 4-year test period, the radiological department of the local university hospital offered to perform neck-MRI in the context of this research study. Access to the MRI unit was therefore facilitated and no additional costs were incurred either for the forensic department, the victim or the prosecutor’s office. Nevertheless, we noticed that very few victims of survived strangulation were actually examined by a neck-MRI during the test period. Surprised by this observation, we decided to review the procedure and evaluate the situation. Therefore, the aims of this study were 1) to understand the limited use of MRI examination of the neck by forensic experts, 2) to identify medicolegal parameters that should be used as indication for neck-MRI in the future at our institute, and 3) to evaluate the results of the neck-MRIs performed.

## Material and methods

We retrospectively reviewed the external clinical examination reports from all victims of a survived strangulation examined in our institute between 1 May 2009 and 31 May 2013.

### Inclusion and exclusion criteria

All the victims of strangulation with compression of the neck were included regardless of the method of strangulation (manually or using a strangulation tool, such as a rope).

Victims of assault to the neck without strangulation, such as kicks, firearms, sharp objects (glass fragments, bottles) were excluded.

### Data collection

Two categories of data were extracted: 1) forensic data, extracted from the medicolegal reports compiled by forensic pathologists and 2) radiological data, extracted from the radiological reports on the neck-MRI, performed by board-certified clinical radiologists who studied the existing literature on interpretation of neck-MRI in strangulation victims.

#### Forensic data

Characteristics of the victims (gender and age), the delay between the assault and the external clinical evaluation, whether an MRI was performed or not, the delay between the assault and MRI, and the description of objective lesions (petechiae, bruising and abrasions) were all extracted from forensic data.

Similarly, the description of the subjective symptoms provided by victims were extracted and designated as clinical symptoms, which were categorized as vision disorders, voice disorders, dysphasia and muscular neck pain.

#### Radiological data

Examinations were performed on a 3 Tesla device (Verio; Siemens, Erlangen, Germany) in the MRI unit of the Department of Diagnostic and Interventional Radiology of the University Hospital of Lausanne.

We performed most of the sequences with different weighted-contrast images (T1; T2; T2 fat saturation (Fat-Sat); and short tau inversion recovery (STIR)) proposed by Christe et al. [13] in their study.

The acquired planes were transverse in T1-weighted, T2-weighted, T2-weighted Fat-Sat and STIR, covering the hard palate to the sterno-clavicular joint and coronal plane in STIR from the middle of the tongue to the backside of the neck including the skin. All technical parameters are provided in Table 1.

We extracted all the MRI findings that could be related to the assault from the radiological reports.

**Table 1.** Parameters used for the sequences of the neck-MRI.

Sequences	TR (ms)	TE (ms)	TI (ms)	Slices thickness (mm)	Reconstructed voxel size (mm × mm × mm)
T1 transverse	620	9.8	-	3	0.6 × 0.6 × 3
T2 transverse	5 000	96	-	3	0.5 × 0.5 × 3
T2 Fat-Sat transverse	6 590	90	-	4	0.6 × 0.6 × 4
T2 STIR coronal	6 460	80	190	4	0.9 × 0.9 × 4
T2 STIR transverse	6 750	80	210	4	0.9 × 0.9 × 4

TR = time repetition; TE = time echo; TI = time inversion; T1 = T1 weighted; T2 = T2 weighted; T2 Fat-Sat = T2 fat saturation weighted; T2 STIR = T2 short tau inversion recovery weighted.

## Results

### Forensic findings

The forensic pathologists evaluated 112 victims of survived strangulation (male/female: 19/93, age: median 48 years, range 5–85 years) during the study period. The time interval between assault and clinical examination ranged from 3 h to 336 h (median 24 h). Four groups of victims were observed: Group 1 (*n* = 8) presented petechiae and related clinical symptoms. Group 2 (*n* = 76) presented bruising, abrasions without presence of petechiae, among whom related clinical symptoms were presented in 66 victims. Group 3 (*n* = 19) did not present any objective lesions but only related clinical symptoms. Group 4 (*n* = 9) presented neither objective lesions nor related clinical symptoms.

Thirteen victims were proposed for MRI examination by the forensic pathologists. Eleven MRI were performed, one victim refused the examination during the clinical evaluation and one victim was not presented to the radiological department.

For Groups 1 and 2, four and nine MRI examinations were proposed, respectively. No MRI examinations were proposed by the forensic pathologists for Groups 3 and 4.

**Figure 1.**
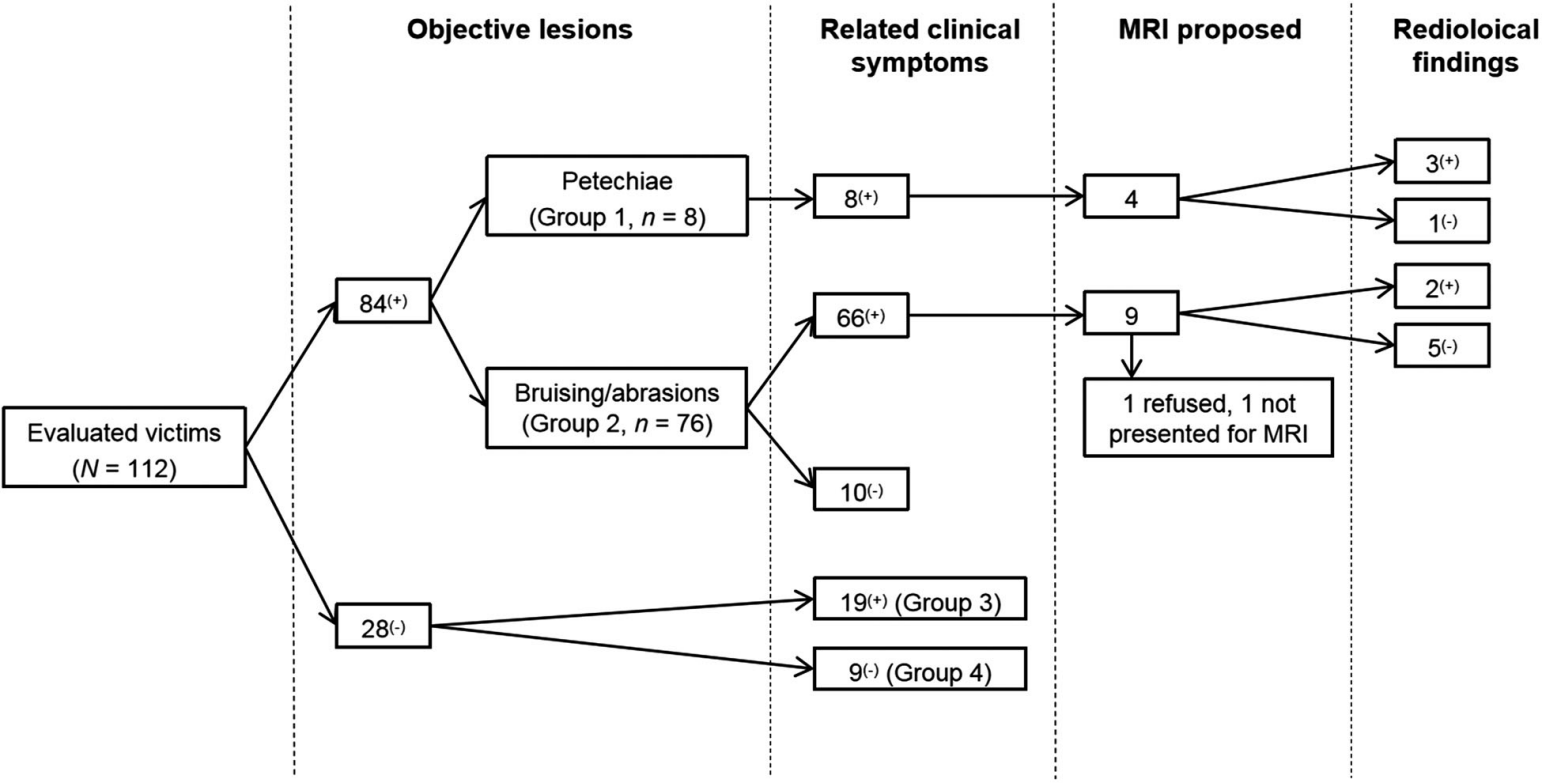
Assignment of the evaluated victims, according to the presence of objectives lesions and related clinical symptoms.

The number of examined victims and their assignment to each of the groups, as well as the performance/non-performance of MRI is described in Figure 1.

The time interval between the clinical examination and MRI ranged from 3 h to 96 h (median 48 h) and the interval between the assault and MRI ranged from 24 h to 120 h (median 48 h).

### Radiological findings

From the 11 MRI examinations performed, there were five cases with positive radiological findings (Table 2). In Group 1, three victims with positive radiological findings were observed; and two victims were observed from Group 2 (Figure 1).

**Table 2.** The different cases, with a description of the radiological findings and the associated forensic clinical findings.

Case No.	Radiological findings	Forensic clinical findings	
		Objective signs	Clinical symptoms
Case 2	Blood serum level in the glottis space + oedema left vocal cord	Petechiae + bruising	Vision disorder Dysphagia Voice disorder Neck pain
Case 4	Haemorrhage in the left platysma muscle + left sternocleidomastoid muscle + left superior jugulocarodit ganglion	Bruising	Dysphagia Voice disorder Neck pain
Case 17	Haemorrhage in the left sternocleidomastoid muscle + haemorrhage in the left platysma muscle + haemorrhage of the left vocal cord	Bruising	Vision disorder Dysphagia Voice disorder Neck pain
Case 22	Subcutaneous oedema + haemorrhage in the right platysma muscle	Petechiae + bruising	Vision disorder Neck pain
Case 62	Haemorrhage in the left sternocleidomastoid muscle	Petechiae + bruising	Vision disorder Dysphagia Voice disorder Neck pain

There were one or more radiological findings per case.

**Figure 2.**
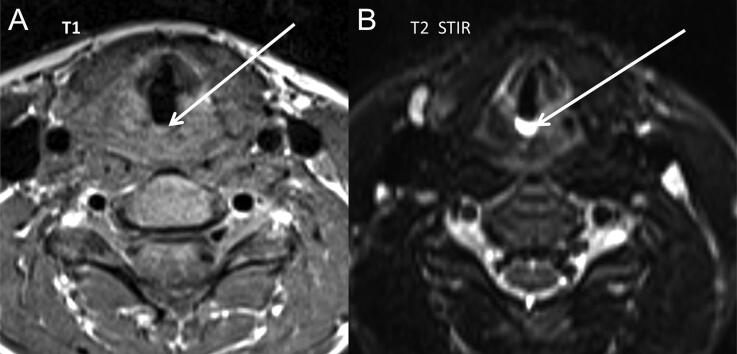
Case 2, transverse plane. Blood serum level in the glottis space (white arrows: (A) isosignal in T1 weighted and (B) hypersignal in T2 short tau inversion recovery (STIR) weighted).

**Figure 3.**
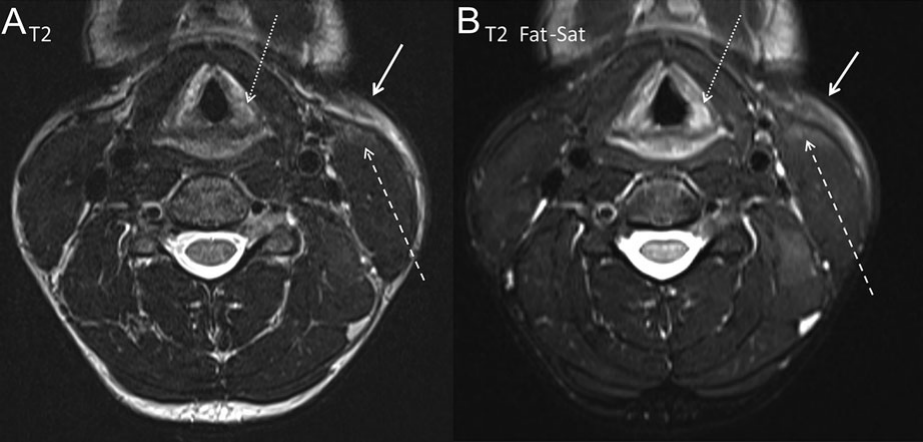
Case 17, transverse plane. (A) Haemorrhage hypersignal in T2 weighted and (B) in T2 fat saturation (Fat-Sat): in the left platysma muscle (white arrows), in the left sternocleidomastoid muscle (dashed arrows) and in the left vocal cord (dotted arrows).

**Figure 4.**
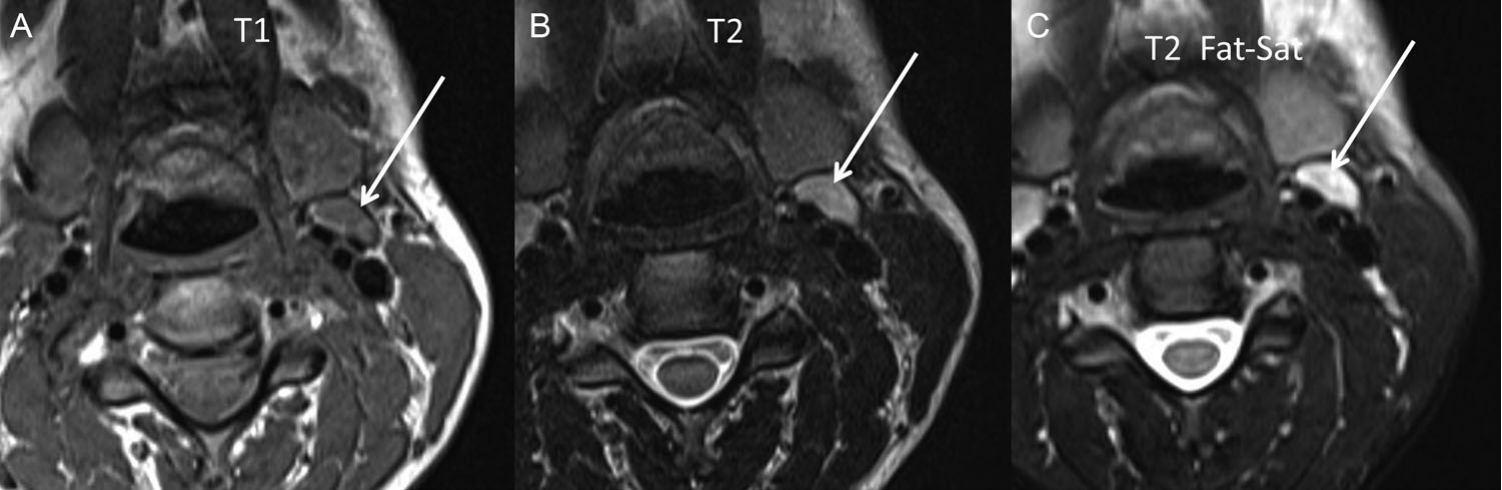
Case 4, transverse plane. Haemorrhage in the left superior jugulocarodit lymph node (white arrows): (A) Isosignal in T1 weighted; (B) hypersignal in T2 weighted and (C) T2 fat saturation.

Blood serum level in the glottis space (Figure 2), haemorrhage of the vocal cord (Figure 3), haemorrhage in the platysma muscle (Figure 3), haemorrhage in the sternocleidomastoid muscle (Figure 3), superior jugulocarotid ganglion haemorrhage (Figure 4) and subcutaneous tissue haemorrhage were described by the board-certified radiologists (Table 2).

## Discussion

The purposes of our study were to understand the limited use of neck-MRI by forensic pathologists in cases of survived strangulation, to identify clinical parameters that should be used as an indication for proposing MRI examinations to victims, and to evaluate the results of the neck-MRIs performed.

Our results showed that of the 112 assessed victims, neck-MRI was only proposed in 13 cases (12%), indicating that the use of MRI evaluation was very low.

Positive radiological findings were observed in 45% (5/11) of the victims who underwent the MRI examination. The different radiological lesions described by Yen et al. [10] and Christe et al. [13] were identified in the cases of neck-MRI.

In Group 1, despite the presence of petechiae which was a criterion for proposing an MRI examination, four cases were not MRI-documented (Figure 1).

In Group 2, 66 victims presented the same clinical symptoms (bruising and clinical symptoms). However, an MRI examination was only proposed in nine cases, from which two victims presented positive radiological findings (22%). If we extrapolate based on this statistic, positive radiological findings may potentially have been visible in 12–13 additional cases in this group, if an MRI examination had been performed.

We were astonished to observe that victims presenting the same clinical symptoms and belonging to the same group (Group 1 or 2) were not proposed for an MRI examination. This finding leads to the suspicion that forensic pathologists did not always adhere to the clear clinical parameters requiring proposal of an MRI.

We can assume that the majority of forensic pathologists function in a binary way when performing their evaluation: presence/absence of petechiae or other objective signs (bruising or abrasions of the skin) localized in the neck or on the face and relevance of subjective symptoms (voice disorder, visions disorders, dysphagia, pain in neck muscle), but without the need to integrate an MRI examination of the neck. For example, in the 10 cases belonging to the Group 2 (Figure 1) that presented objective signs (bruising) without clinical symptoms, the presence of objective lesions seemed to have no influence in the decision whether to propose an MRI examination, even if it were decided to include these cases to compare our results with those of preliminary studies [9, 10, 13, 14].

To explain the very few MRI proposals, we can also consider the hypothesis that there is reluctance to accept the addition of a new examination into a well-run medical workflow. This may be especially significant when the examination and its added value is not fully understood or not yet supported by enough scientific literature, or where knowledge of specialized literature outside of their field of expertise is lacking. Groups 3 and 4 are certainly in this dynamic, and there was no doubt for forensic pathologists when reaching their conclusion: these cases did not require a further examination in their opinion. As the literature shows, internal lesions causing symptoms such as voice disorder could be visualized in MRI even without presence of objective lesions, however [15–18]. We therefore believe that it would have been useful to propose an MRI examination to victims of Group 3 and even for Group 4.

Despite the few cases evaluated by MRI, this study had an impact on the forensic pathologists of our institute. Firstly, it reinforced the potential of MRI to depict various radiological findings in the inner soft tissues that are not visible during an external evaluation, in addition to their clinical examination. Secondly, it demonstrated that there is a lack of uniformity in the way in which victims are assessed and consequently the way decisions regarding additional examinations are made. Hence, there is a strong need to review the procedure for proposing MRI to victims.

The first step was to define new inclusion criteria. One approach was to ask the question: if an objective lesion is present, is a neck-MRI useful? In the forensic report, if the forensic pathologist describes visible lesions, the addition of radiological findings is not likely to change this report dramatically, since radiological findings are not currently validated to confirm the life-threatening status of injuries, despite the concept of stratification of sensible anatomical zones as proposed by Christe et al. [13]. But the provision of images that can objectify the presence of internal lesions remains an important element.

For victims who do not present objective signs, we can assume that radiological findings may influence the final report by describing inner lesions not assessable during the clinical evaluation. Thus, it would also be useful to be able to propose for each victim without objective lesions an MRI examination, in order to prove the compatibility of the elements reported by the victims.

However, an important point to consider as the evaluation study being completed, was to find new financial resources for covering the cost of this radiological examination. Thanks to the radiological findings of our study, which convinced prosecutors of the usefulness of the neck-MRI in addition to a clinical evaluation for victims, they decided to pay for the exams in certain cases.

In our country, the price of an MRI examination depends upon factors such as the duration of the examination. Taking advantage of this constraint, we also decided to update the sequences proposed over a decade ago by Christe et al. [13, 14] with sequences used regularly in routine clinical practice for neck-MRI. These sequences are faster to perform and more modern, but also retain the various planes and coverage required for reliable assessment of internal injuries related to strangulation. The addition of sequences for the brain reflecting cerebral anoxia [16–18], the symptoms of which may appear later [19–23], were also discussed, but unfortunately the additional time required for those sequences is excessive and would increase the cost of the MRI examination by more than is acceptable for prosecutors.

### Limitation

The major weakness of this study is that it was impossible for us to objectively identify the reason why this new tool was not well accepted by forensic scientists. At the time of the implementation of this study, the forensic pathologist in charge of it was convinced that the explanation of the advantages of MRI for soft tissue evaluation, the scientific articles made available, and the facilitated access to the MRI device through agreements with the radiology departments, would be sufficient to convince his colleagues to offer this examination to the survived victims of strangulation. The absence of a written procedure validated by the medical hierarchy and the lack of follow-up of this study also had an effect (not objectively assessable) on the limited neck-MRI proposed and the lack of uniformity in victim assessment.

## Conclusion

Despite the small number of cases included in this study, we were able to critically reflect on the procedures and inclusion criteria for the proposal of MRI examinations in cases of suspected strangulation. Furthermore, we have demonstrated that MRI is a useful tool for the evaluation of victims of survived strangulation and should be proposed in addition to clinical examinations. Finally, to ensure the consistency of clinical forensic reports in cases of suspected strangulation and the inclusion of all useful examinations, strict and clear indications and procedures for forensic pathologists need to be established.

## References

[1] Brinkmann B. [Asphyxia]. In: Brinkmann B, Madea B, editors. Handbuch Gerichtliche Medezin. Berlin (Germany): Springer; 2004. p.761–794. German.

[2] Brinkmann B, Püschel K. Ersticken-Fortschritte in der Beweisfürhrung. Berlin (Germany): Springer; 1990. German.

[3] Knight B, Saukko P. Knight’s forensic pathology. 4th ed. London (UK): CRC Press; 2015. Chapter 15, Fatal pressure on the neck; p. 369–397.

[4] Spitz WU, Fischer RS. Medicolegal investigation of the death. 3rd ed. Springfield (IL): Charles C. Thomas; 1993, p. 829.

[5] McClane GE, Strack GB, Hawley D. A review of 300 attempted strangulation cases. Part II: clinical evaluation of the surviving victim. J Emerg Med. 2001;21:311–315. 11604295 10.1016/s0736-4679(01)00400-0

[6] Plattner T, Bolliger S, Zollinger U. Forensic assessment of survived strangulation. Forensic Sci Int. 2005;153:202–207. 16139111 10.1016/j.forsciint.2004.09.106

[7] Schrag B, Vaucher P, Bollmann MD, et al. Death caused by cardioinhibitory reflex cardiac arrest—a systematic review of cases. Forensic Sci Int. 2011;207:77–83. 20961719 10.1016/j.forsciint.2010.09.010

[8] Schrag B, Mangin P, Vaucher P, et al. Death caused by cardioinhibitory reflex: what experts believe. Am J Forensic Med Pathol. 2012;33:9–12. 22442830 10.1097/paf.0b013e3181db7efd

[9] Yen K, Thali MJ, Aghayev E, et al. Strangulation signs: initial correlation of MRI, MSCT, and forensic neck findings. J Magn Reson Imaging. 2005;22:501–510. 16142698 10.1002/jmri.20396

[10] Yen K, Vock P, Christe A, et al. Clinical forensic radiology in strangulation victims: forensic expertise based on magnetic resonance imaging (MRI) findings. Int J Legal Med. 2007;121:115–123. 17206435 10.1007/s00414-006-0121-y

[11] Harnsberger HR, Osborn AG, Jeffrey SR. Diagnostic and surgical imaging anatomy: brain and head neck, spine. 1st ed. Salt Lake City (UT): Amirsys, Inc; 2006.

[12] Baert AL, Reiser MF, Hricak H, et al., editors. Head and neck cancer imaging. 2nd ed. Heidelberg (Germany): Springer;2006.

[13] Christe A, Thoeny H, Ross S, et al. Life-threatening versus non-life-threatening manual strangulation: are there appropriate criteria for MR imaging of the neck? Eur Radiol. 2009;19:1882–1889. 19283386 10.1007/s00330-009-1353-2

[14] Christe A, Oesterhelweg L, Ross S, et al. Can MRI of the neck compete with clinical findings in assessing danger to life for survivors of manual strangulation? A statistical analysis. Leg Med (Tokyo). 2010;12:228–232. 20630784 10.1016/j.legalmed.2010.05.004

[15] Subramanian M, Hranjec T, Liu L, et al. A case for less workup in near hanging. J Trauma Acute Care Surg. 2016;81:925–930. 27537511 10.1097/TA.0000000000001231

[16] Kalita J, Mishra VN, Misra UK, et al. Clinicoradiological observation in three patients with suicidal hanging. J Neurol Sci. 2002;198:21–24. 12039659 10.1016/s0022-510x(02)00056-4

[17] Singhal AB, Topcuoglu MA, Koroshetz WJ. Diffusion MRI in three types of anoxic encephalopathy. J Neurol Sci. 2002;196:37–40. 11959154 10.1016/s0022-510x(02)00019-9

[18] Matsuyama T, Okuchi K, Seki T, et al. Magnetic resonance images in hanging. Resuscitation. 2006;69:343–345. 16458413 10.1016/j.resuscitation.2005.08.003

[19] Hori A, Hirose G, Kataoka S, et al. Delayed postanoxic encephalopathy after strangulation. Serial neuroradiological and neurochemical studies. Arch Neurol. 1991;48:871–874. 1898266 10.1001/archneur.1991.00530200113030

[20] Malek AM, Higashida RT, Halbach VV, et al. Patient presentation, angiographic features and treatment of strangulation-induced bilateral dissection of the cervical internal carotid artery. Report of three cases. J Neurosurg. 2000;92:481–487. 10701540 10.3171/jns.2000.92.3.0481

[21] Kiani SH, Simes DC. Delayed bilateral internal carotid artery thrombosis following accidental strangulation. Br J Anesth. 2000;84:521–524. 10.1093/oxfordjournals.bja.a013484 10823110

[22] Imamura K, Akifuji Y, Kamitani H, et al. Delayed post anoxic encephalopathy with visual field disturbance after strangulation: a case report. Brain Nerve. 2010;62:621–624. 20548123

[23] Sethi PK, Sethi NK, Torgovnick J, et al. Delayed left anterior and middle cerebral artery hemorrhagic infarctions after attempted strangulation: a case report. Am J Forensic Med Pathol. 2012;33:105–106. 21512390 10.1097/PAF.0b013e3182198672

